# Operative Dentistry

**Published:** 1880-07

**Authors:** Isaac J. Wetherbee


### ARTICLE II.

### *Operative Dentistry.*

BY ISAAC J. WETHERBEE, D. D. S.

A compilation of the rules and systems of practice of the "ten thousand" (more or less) practitioners of dentistry in the United States would present a condition of things that would awaken the greatest possible surprise, so diverse are the views of dentists as to methods to be employed to obtain a specific end.

It there was *unity* in this *diversity* a satisfactory personal record would ensure a complete jurisdiction. In the absence of such record we are forced to the conclusion that there is, at the present time, a dearth of scientific knowledge requisite for a skillful practice of dental surgery—knowledge of the true character and functions of dental tissues obviously required—the proper methods of procedure to converse, to the fullest extent, their usefulness.

It appears that late events—events characteristic of the age in which we live—have unsettled, or at least disturbed, the convictions of many, which had matured from long and careful observation. Why this sudden change? One might reasonably suppose that a *messenger of light* from a higher plane had at least condescended to give us a "new dispensation."

Let us in a few brief words examine the *salient points* of this "new departure," and see if there is anything really "new" about it, and if so, in what it consists. It is acknowleged by the chief apostle and other expounders of the "new departure" that in *well organized teeth* gold should be employed as a suitable material. In the "Dental Cosmos" for July, on page 376, we have the *true inwardness* of this latter-day adventure set forth with an unction that is exceedingly refreshing. Hear what it says:

"We desire that you should observe *how little*, *how very little*, we have asked that you should deviate —at first—in that which is practice from that which is usual practice."

"We say, *fill all you can and do*, SATISFACTORILY WITH GOLD! *It is so stated in the bond!* Can you in turn ask anything more? Why do you desire to fill such teeth as you cannot fill 'SATISFACTORILY' with gold with that material?"

Is there anything "new about that?"

"But the truth is that the 'true inwardness' of the 'new departure' is a very different thing from this. Its design is to teach a principle that shall indicate lack of knowledge as the cause of '*failure in operations*,' instead of attributing it to defective manipulation."

This audacious assumption! this begging of the whole question, especially after ceding the fundamental principles which underlie dental practice as generally understood, is quite enough to make angels weep, if they have *tears to spare* for the washing away o such folly?

We are *advised* to fill all we can satisfactorily with gold. Why? Because gold makes a good and durable filling in the majority of teeth, notwithstanding the *assumed want of compatibility of gold with dentine.* How shall we determine the compatibility of gold with dentine? Because teeth again decay after having been well filled; and from the same cause or causes which produced the first lesion are we to legitimately infer that the chief factor in this newly recurring disaster is to be charged to want of compatibility. Oh consistency, thou art a jewel!

Again, "it is the *little*, yea, *very little* deviation that they have asked at *first* from that which is usual practice." Now, why all this? If gold is radically wrong in the *one case* for the want of compatability, why not in another? And how is it about the *little* departure? Why not away with it at once and make it contraband? The incongruity and fallacy of such teaching is quite enough to indicate a relish for the leeks and onions of Egypt.

The enunciation of the plausible theory of chemico-electro galvanic action produced by gold being in opposition to dentine, and producing decay, has had its day of small sayings, without proofs, and is seeking a retired place for silent *interment*, for the reason that it could not find either godfather or reliable god-mother as sponsors to assume the responsibility of its future well being, as it had no established scientific basis on which to depend.

We are informed that the *true inwardness* of the "new departure" is a very diffeient thing from this. Its design is to teach us *how little we know* of the cause of "failure" in operations. This gratuity on the part of one who was possessed of this specific knowledge for eighteen or twenty years but retained it within his own bosom until recently, is quite overwhelming. At least it seems that his bowels of compassion for suffering humanity were not moved until the fullness of time approved by himself had arrived

Is it not true that dentists in their practice are electic? Have they not, as a general thing, filled such teeth with gold when the quality of the tooth indicated its use? Have they not used amalgam, when in their judgement, the usefulness of the tooth could best be preserved thereby? Have not the oxychlorides and tinfoil had due prominence in the economy of dental practice? When doubt has existed as to saving the teeth by the employment of either of the above materials has not a resort to extraction been a reasonable practice?

It is true that many teeth have been sacrificed in the past, and will be in the future, for lack of knowledge and because of a prudishness as to reputation. Dead men tell no tales (not true, however,) and their escape from implied indiscretion, by reason of the failure of some operations, is a personal satisfaction. It is reasonable to suppose that the employment of the above mentioned materials is due to a conviction that a tooth may be preserved longer and be more useful, and this from a *knowledge* of the *relative conditions* of the case.

It is a generally well known fact that "gutta percha" will preserve a tooth as long as it shall remain intact. Six months or a year will suffice to disintegrate such fillinings the teeth of patients whose oral secretions are viscid. In other and divers cases these fillings will be worn away by attrition within a comparatively short space of time. They must be *frequently repaired.* Each sitting for repairs is an additional expense to the patient, and in the lapse of one decade will aggregate a larger expense than would have occured provided they had been filled with gold in the first instance, to say nothing of the loss of time. Let us not forget or be unmindful of the frequent repair the "new departure" system subjects patients to.

It is my intention in this report to voice the general sentiment of dentists so far as I have been able to obtain their views; and give, as far as possible, an opinion which many years of intelligent practice has cnnfirmed. I believe that no practical man expects to save all the teeth which he fills for a definite period. It is understood that although cavities in the teeth may be filled, *and filled perfectly*, we have little, if any, control over the causes which first produced the local destruction of the dental tissues. Decay may sieze any other portion of the tooth, or, indeed may waste away the enamel and dentine in proximation to the gold.

Again, other teeth, which were not decayed when the above cases were treated, are found, after three or four years decayed, not by the apposition of gold thereto, but as the result of viscid secretions or fluids, and perhaps for a lack of proper care of the teeth. In such cases we have no use for the hypothetical; the evidence is clear and conclusive.

So far as my knowledge and observation extend, there are few converts to the so-called "new departure."

I deem it proper in this report to give a brief summary of what may be considered general practice. The *contourist* claims that in good tissue the lost portions should be restored to nearly or quite the normal size, believing that nature, in her economy, is to be followed in order to secure the best practical results. My convictions and practice are in perfect accord with the theory above expressed. Operations of like character performed over twenty years since, are good to-day, and conclusively demonstrate the practicability of this style of practice.

We have the *radical separatist*, who, with file and chisel cuts away both good and poor tissues alike, making no discrimination, leaving the teeth in a sadly demoralized condition. The excuse offered for this is greater facility in operating, larger degree of cleanliness and less liability to decay. In poor teeth this practice is quite justifiable; indeed the condition of the teeth as to quality makes it necessary, as the teeth can only be saved, if preserved at all, by this method. But why extend this practice to good and well organized teeth?

We have next in order the *more moderate separatist*, who is careful to secure a sufficient general separation; but is also careful to give a much wider space on the lingual approximate surfaces, for the purpose of making the space self cleansing, thereby retaining the good appearance of the tooth. This practice so far as conditions may indicate, is to be approved and commended. The surfaces in such cases should be well polished

Gold in the form of *pellets*, if non-cohesive, is well adapted for use at the cervical wall, and in large or small crown cavities cylinders are admissible; by some they are preferred. For the general use of cohesive foil I prefer ribbons, made by folding a sheet of No. 4 so as to make eight thicknesses, and then cut into such sizes as the case shall require. Gold in this form is adapted to cases where but little room for convenient operating is obtained, as it is very portable. The method of impacting, etc. etc., are too well known to be rehearsed in this paper.

Let us consider the claims which some of the "bleachants" now in use have upon our confidence. Chloride of lime, or, more properly speaking, *chlorinated lime*, has been used for bleaching discolored teeth. It has, however, fallen into disuse, for the reason that, although at first its use was satisfactory, future results did not justify its application. Chloride of sodium is open to like objection, from the effect produced upon the calcareous portion of the dentine, as well as upon the soft tissue. A grayish-yellow shade will appear within a short time or at best within a few months.

Strong *spirits of camphor* is an excellent bleachant; it is very effective in a short space of time. It dissolves the pigment within the cells, as well as permeating the hard tissue, leaving no bad results. So much of the dentine should be removed as may be safe to do. The rubber dam should be in position, and the spirits of camphor applied for such time as may be requisite to attain the end desired. Then fill the *root* with gold in all cases. Fill the cavity with great care and thoroughness, and you will be well pleased with your success.

There has not transpired within the past year anything *new* with reference to the best mode of *extracting teeth*, a more effectual method of treating cases of *salivary calculus*, nor a surer method of disposing of *alveolar abscesses.* To extract an abscessed tooth, and then replace it, for the purpose of removing the difficulty, is objectionable practice and should, in my opinion, be abandoned.

[The majority of the section would respectfully dissent from the statement that all roots of the anterior teeth should be filled with gold, believing as they do, that other materials may be used for the purpose more easily and with equal success.

Also, they would state that they regard the replantation of teeth, for the cure of alveolar abscess, as good practice in certain extreme cases.]—*Transactions of Am. Dent. Association.*

